# Factors and framing effects in support for net zero policies in the United Kingdom

**DOI:** 10.3389/fpsyg.2023.1287188

**Published:** 2023-12-15

**Authors:** Wouter Poortinga, Lorraine Whitmarsh, Katharine Steentjes, Emily Gray, Sophie Thompson, Rachel Brisley

**Affiliations:** ^1^Centre for Climate Change and Social Transformations (CAST), School of Psychology, Cardiff University, Cardiff, Wales, United Kingdom; ^2^Welsh School of Architecture, Cardiff University, Cardiff, Wales, United Kingdom; ^3^Centre for Climate Change and Social Transformations (CAST), Department of Psychology, University of Bath, Bath, United Kingdom; ^4^School of Psychology, Swansea University, Swansea, Wales, United Kingdom; ^5^Ipsos UK, London, United Kingdom

**Keywords:** net zero, climate change, policy support, framing effects, perceived fairness

## Abstract

Achieving ambitious carbon reduction targets requires transformative change to society, with behaviour change playing an important role. Climate change mitigation (‘net zero’) policies are needed to accelerate and support such behaviour change. This study examined factors and framing effects in public support for net zero policies in the United Kingdom (UK), making use of a large probability sample (n_total_ = 5,665) survey conducted in August 2021. It found that net zero policies are widely supported, with only taxes on red meat and dairy products being supported by less than half of the UK public. Climate worry and perceived fairness were the strongest and most consistent predictors of policy support for net zero policies. The results further suggest that support for net zero policies can be increased by emphasising the co-benefits of the policies, in particular where they are beneficial for health. However, the framing effects were very small. In contrast, public support for net zero policies is lower when potential lifestyle and financial costs are mentioned. This suggests that perceived fairness of the distribution of costs and lifestyle implications of policies are crucial for building and maintaining support for net zero.

## Introduction

### Background

Climate change poses profound risks to humans, animals and ecosystems, ranging from rising sea levels and extreme weather to spread of disease and crop failures (IPCC, 2021). In order to mitigate these risks, and limit global warming to no more than 2°C above pre-industrial levels, carbon emissions from human activities need to be substantially reduced and reach ‘net zero’ by 2050 (IPCC, 2022). Net zero means reducing all possible carbon emissions and then offsetting any remaining emissions (Fankhauser et al., 2022). The United Kingdom (UK) was the first country to implement legally-binding targets to reduce carbon emissions, with a target of reaching net zero by 2050 and 78% reduction in emissions by 2035 (UK Government, 2008, 2019). This will however require transformative change, with behaviour change playing a central role in that transformation. Most of the measures needed to reach net zero require at least some behavioural change, including the adoption of low-carbon technologies and wider lifestyle changes (Carmichael, 2019; Climate Change Committee, 2020; IPCC, 2022).

Public concern about climate change has been rising gradually over the past 10 years, and now large majorities in most countries say they are worried about climate change (Poortinga et al., 2019; Ipsos, 2023). In addition, people recognise the urgency with which climate change needs to be addressed and are also aware of the need for change (Steentjes et al., 2021). However, environmental action remains largely limited to small-scale lifestyle changes, such as recycling (Whitmarsh, 2009; Ipsos, 2023). More impactful actions are needed to substantially reduce greenhouse gas emissions, including avoiding flying and driving and adopting plant-based diets (Wynes and Nicholas, 2017; Ivanova et al., 2020). Many of these impactful actions do not only reduce greenhouse gas emissions, but also provide benefits for health, the economy and the environment. A move towards more plant-based diets not only has the potential to reduce food-related greenhouse gas emissions by up to 70 percent, it also can help to reduce global mortality by up to 10 % (Springmann et al., 2016). There are also clear health benefits associated with a shift to lower-carbon transport modes. A shift to electric vehicles, public transport and active travel modes reduces congestion, air and noise pollution, and road accidents (Smith et al., 2016), with additional health benefits of increased exercise from walking and cycling (Shaw et al., 2014).

While these behavioural changes can help achieve wider sustainability goals and improve human wellbeing (Creutzig et al., 2022), they can be costly; and many individuals report substantial barriers (e.g., costs, inconvenience, etc.) to environmental action. For example, switching to a more plant-based diet provides health benefits, but may also involve paying more for ingredients or learning new food preparation skills (Whitmarsh et al., 2021). Meaningful policies are therefore needed to enable the behaviour change required to reach net zero. Regulation, economic (dis)incentives, and changes to infrastructure can all be used to promote low-carbon and discourage high-carbon options (Grubb et al., 2020; House of Lords, 2022). Yet, these policies are often controversial and have sparked high profile protests in some cases (Howarth et al., 2020). Indeed, several policies to enable low-carbon behaviour to have met with considerable public opposition. Low Traffic Neighbourhoods (LTN) that use modal-filters to restrict through traffic in residential areas have proven to be controversial (Aldred, 2019; Campbell, 2023), as has the implementation of new cycle lanes as part of the Emergency Active Travel Fund that was designed to promote walking and cycling in response to the COVID-19 pandemic (Morton, 2020; Campbell, 2023). Similarly, policies to reduce red and processed meat consumption lack widespread support (Pechey et al., 2022), not least because there are perceived as being not particularly effective (Steentjes et al., 2021). Furthermore, meat consumption remains deeply embedded in social and cultural norms (Sievert et al., 2022), and meat curtailment policies are often seen as an infringement on freedom of choice (Michielsen and van der Horst, 2022).

Understanding the basis of public support for and opposition to net zero policies is essential for their effective design and implementation. Previous research demonstrates that public participation in environmental policy design can improve its efficacy and acceptability, since engaging with diverse groups can shed light on the workability and perceived fairness of policies and increase trust in and legitimacy of the policy process (Stern and Dietz, 2008; Höppner and Whitmarsh, 2012). With regards to climate change policies, given the scale of disruption these may bring to lifestyles and the economy, it is essential to foster public buy-in for these changes but also to understand why different policies may be accepted or rejected and how support may be fostered. Understanding public support for net zero policies can also create the political mandate needed for ambitious climate policies, emboldening leaders to act in line with voter preferences (Howarth et al., 2020).

#### Factors in support for net zero policies

The literature suggests that people are more likely to support environmental policies if they are seen as effective in addressing the problem at hand (e.g., reducing air pollution, congestion etc) and/or provide clear benefits for society as a whole or for oneself personally (Dieplinger and Fürst, 2014; Schwirplies et al., 2019; Schuitema et al., 2020). At the same time, non-coercive ‘pull’ measures, such as subsidies, financial support and information provision are supported more than coercive ‘push’ policies, such as regulation and taxes (Eriksson et al., 2008; Drews and van den Bergh, 2016). In particular, information provision, which fully maintains individual choice and generally does not impose costs on the individual, is seen as inoffensive and therefore supported across the board (Poortinga et al., 2022). Perceptions of coerciveness, however, depend on people’s personal circumstances. People are less likely to support a policy if they think they will be personally affected it. For example, in particular car owners perceive road pricing as an infringement on freedom, and therefore are more likely to oppose such measures (Jakobsson et al., 2000; Fujii et al., 2004; Hammar and Jagers, 2007).

Another key factor in policy acceptance is perceived fairness. People are more likely to support climate policies if they think they are fair. Indeed, perceived fairness is often a stronger predictor of climate change policy support than perceived effectiveness (Ejelöv and Nilsson, 2020; Bergquist et al., 2022). Perceived fairness has distributive and procedural components. Perceived distributive fairness involves perceptions of how costs and rewards of a policy are shared across different groups, while perceived procedural fairness refers to the fairness of processes by which decisions are made, in particular whether the decision-making process is transparent and takes into account views of everyone affected (Greenberg, 1986). The acceptance of and support for environmental policies are dependent on both perceived distributive fairness and perceived procedural fairness (Besley, 2010; Schmöcker et al., 2012; Dreyer and Walker, 2013; Kim et al., 2013; Liu et al., 2020). People are more likely to support policies that target those who are the most responsible and do not disproportionately affect vulnerable or (economically) disadvantaged groups. That is, people are more supportive of policies that are proportional to current and historical emissions, i.e., adopt a ‘polluter pays’ principle, and take account of someone’s ‘ability to pay’ (Cai et al., 2010; Bechtel and Scheve, 2013), as well as polices that have consulted all groups that are likely to be affected and are considerate of them (Climate Assembly UK, 2020). The extent to which policies are seen as fair and thus supported does however vary across different socio-demographic groups, such as income, age and place of residence (Player et al., 2023). This may be due to different needs, opportunities and abilities, which is likely to impact upon the extent the different groups are affected by the policy (Player et al., 2023).

Individual values and attitudes are important factors in policy support (Bouman et al., 2020). Problem perception has been identified as a key determinant of policy support in theories such as the Value-Belief-Norm model of pro-environmental behaviour (Stern, 2000). Consequently, climate change concern is a strong driver of climate policy acceptance (Bergquist et al., 2022). Furthermore, environmental values are linked to lower opposition to ‘push’ policies, like congestion charging (Eliasson and Jonsson, 2011) and carbon taxes (Eriksson et al., 2008), and political orientation predicts climate policy support in many countries (Poortinga et al., 2019). In the US, for example, right-wing orientation is associated with lower support for publicly-financed climate policies (Ziegler, 2017).

Taken together, evidence shows both personal factors (e.g., socio-demographics, climate worry, political values) and policy-specific evaluations (e.g., perceived fairness) shape policy acceptance. However, while studies have examined predictors of specific climate policies or class of policy (e.g., carbon taxation), little work has compared the predictors of a diverse range of net zero measures that span sectors (transport, food, finance, etc.) and instrument type (e.g., regulation, taxation). This limits the comparability and utility of studies, and is an important evidence gap we address in the current study.

#### Framing effects in support for net zero policies

While support for climate policies may vary between different groups, research shows that the way in which policies are communicated or ‘framed’ can also shape support. Framing is how an issue or action is presented, emphasising certain aspects of an issue, often with the intention to influence how an audience perceives it (Lockwood, 2011). In some instances, communicating a policy’s co-benefits, for example for health or finances, may boost policy acceptability. Positively framed health messages can make climate change appear local, short-term, and personal, increasing policy acceptability (Rossa-Roccor et al., 2021). Co-benefits, such as safety, health, or job creation, have also previously increased policy support (Jennings et al., 2020; Dechezleprêtre et al., 2022), and participatory methods such as the UK Climate Assembly UK (2020) have been cited as opportunities to explore policy co-benefits that the public otherwise may not consider. Since people’s preferences for co-benefits vary in line with their personal values, tailoring co-benefit framing to the specific populations may boost acceptability (Whitmarsh and Corner, 2017). Overall, however, framing effects on policy support tend to be small (Bernauer and McGrath, 2016), with some arguing that citizens are overexposed to competing climate-related ‘frames’ in their everyday lives, making experimentally manipulated framing effects challenging to detect and unreliable (Fesenfeld and Rinscheid, 2021). Nevertheless, framing studies shed light on how policies that are designed to achieve climate goals can be communicated in ways that focus on other societal goals and thus achieve wider public support beyond committed green voters. This is arguably a critical goal for achieving political and public consensus on the transition to net zero (Giddens, 2009).

#### Aims of the study

This study explores factors and framing effects in public support for climate change mitigation (‘net zero’) policies in the UK. First, the study examines the extent to which eight net zero policies are opposed or supported by the UK public. Second, it assesses the impacts of benefit framing (in terms of climate change, health and economic benefits) on public support for the net zero policies. Third, it examines how personal and policy-specific factors shape policy support for net zero policies. In particular, it examines how socio-demographic factors, climate worry and political values, as well as perceived fairness of the net zero policies, are associated with policy support. Finally, the study explores the impact that potential personal costs (e.g., having to make lifestyle changes, taking on financial costs) may have on support for net zero. This is to examine whether support for net zero policies is conditional on the potential lifestyle or financial implications they may have.

### Methods

#### The net zero living study

The Ipsos-CAST *net zero living* study was conducted between 19 and 25 August 2021, using the Ipsos UK Knowledge Panel (Ipsos & CAST, 2022). The Ipsos UK Knowledge Panel is an online random probability panel consisting of members of the UK public who have been recruited using address-based random probability sampling. Data were weighted for age, gender, region, deprivation quintile, education, ethnicity, and number of adults in the household to make the sample fully reflective of the UK population. In total, 5,665 people aged 16 years or older took part in the survey. The characteristics of the sample are described in Table 1.

**Table 1:** Characteristics of the Ipsos-CAST net zero living study (*n* = 5,665).

	n	%
Gender		
Female	2,906	51.3%
Male	2,717	48.0%
Age		
16–34	881	15.6%
35–54	1,912	33.8%
55 and over	2,872	50.7%
Ethnic background		
White	5,223	92.2%
Other^1^	379	6.7%
Country		
England (excluding London)	3,753	66.6%
England (London)	496	8.8%
Scotland	1,051	18.6%
Wales	215	3.8%
Northern Ireland	150	2.6%
Type of area		
Rural	1,453	25.6%
Urban	4,212	74.4%
Neighbourhood deprivation		
1st (least deprived)	1,422	25.1%
2nd	1,201	21.2%
3rd	1,148	20.3%
4th	1,032	18.2%
5th (most deprived)	862	15.2%
Scales		M (SD)
Climate change worry (scale 1–5)	5,624	3.52 (1.03)
Left–right (scale 1–5)	4,940	2.62 (0.69)
Liberal-Authoritarian (scale 1–5)	5,144	2.74 (0.72)

The percentages do not always add up to 100% due to missing values and rounding; ^1^Asian/Asian British. Black/Black British, Mixed, Multiple or Other ethnic background.

The study focused on eight net zero policies covering the areas of diet, transport, material consumption, home heating and green finance policy, which are all areas where individual level action is needed to reach net zero. It used policies that already exist or are being discussed in the UK, so that survey respondents could easily envisage the policies and thus have or form an opinion about them (Ipsos & CAST, 2022). The study included policies such as subsidies for electric vehicles, which already exist in the UK (UK Government, 2023) and low traffic neighbourhoods (LTNs) that have received widespread media attention (Dudley et al., 2022). Other proposed policies were selected from existing reports, such as the frequent flyer levy discussed at the Climate Assembly UK (2020).

#### Procedure

Respondents were presented descriptions of four out of eight policies and asked several questions about these policies, including their level of support and their views on the fairness of the policies (each policy was assessed by *n* = 2,731–2,887 respondents). The policies were randomly allocated to each respondent. In order to examine the impact of different framings on support for net zero policies, respondents were also randomly presented one of four descriptions of the net zero policies: (1) a ‘neutral’ description, which contained a technical description of the policies as shown in Table 2, (2) a ‘climate change’ framing, presenting the potential climate impact of the policy, (3) a ‘health’ framing, presenting the potential health, safety or general lifestyle impacts of the policy, or (4) an ‘economic’ framing, presenting the potential financial impacts of the policy. All versions contained the neutral description of the policies with added text for the other framings (See Supplementary Information Table SI1).

**Table 2:** Descriptions of the net zero policy (see also Ipsos & CAST, 2022).

Transport		
Creating low traffic neighbourhoods The government may want to reduce the number of vehicles on the road by creating low-traffic neighbourhoods. This is where cars, vans and other vehicles are stopped from using residential roads as shortcuts. This is done by putting some road closures in place using measures such as bollards or planters. Residents are still able to drive onto their street, but it is made more difficult or impossible to drive straight through the area from one main road to the next.	Frequent flyer levies The government may want to replace current tax on flights (Air Passenger Duty) by a tax that increases as people fly more often. People who only fly once in a year could pay no tax, while people who fly several times per year could pay a large amount of tax. This could mean people replace some flights with alternatives, like trains or ferries, or with video conferencing instead of some business travel.	Electric vehicle (EV) subsidies The government may want to subsidise the purchase of electric vehicles for consumers in order to reduce the number of petrol and diesel cars on the road. The government is ending the sale of new petrol and diesel cars by 2030 and encouraging a shift to electric vehicles. Putting in place subsidies, would mean electric vehicles become less expensive to buy than they are now. The money to do this may come from increasing fuel duty on petrol and diesel cars
Home heating	Material consumption	Green finance
Phasing out the sale of gas and coal boilers The government may want to cut down on the use of fossil fuel energy by banning the sale of new gas boilers in the next few years, for example by 2030. This would mean that when homeowners come to replace their boilers, they would need to buy a different sort of heating system, such as an electric heat pump or hydrogen boiler. This may cost more initially but is likely to be cheaper to run in the longer term	Changing product pricing to reflect how environmentally friendly products are The government may want to replace current tax on products by a tax that will vary according to the negative environmental impacts of different products. This would mean products that are produced using high amounts of resources such as energy, water or scarce metals, or products that travel long distances before being sold in a shop would be more expensive than products that are manufactured in more environmentally-friendly ways	Ensuring access to sustainable pension funds The government may want to increase the public’s access to sustainable pension funds. This means that they would increase regulations to ensure that all pension providers include a pension fund option for people to choose from that only used sustainable investments that do not harm people or the planet. This would be the default pension option for the general public, unless they chose to opt out of it.
Food and diet		
Increasing vegetarian/vegan options in public food provisioning The government may want to reduce the amount of red meat and dairy products people eat, by increasing vegetarian and vegan options in all public sector catering. This would mean that meals served in hospital cafés, school canteens, prisons, police and fire stations, council offices, and across the public sector, would need to include a significant proportion of meat-free and plant-based options. It would reduce but not remove meat and dairy from menus, while it would increase the choice of meat/dairy-free alternatives.	Higher taxes on red meat and dairy products The government may want to replace current tax on food products by a tax that will vary according to the negative environmental impacts of different foods. This would increase the price of red meat and dairy products, and reduce the price of certain other foods (e.g., vegetables, bread)	

#### Measures

##### Dependent variables

The main dependent variable in this study was *support for net zero policies*. This was measured with a single item (“To what extent do you support or oppose this?”), with a 5-point response scale (1 “Strongly oppose,” 2” Tend to oppose,” 3 “Neither support nor oppose,” 4 “Tend to support,” and 5 “Strongly support”). The included net zero policies were: creating low traffic neighbourhoods (LTN), frequent flyer levies, electric vehicle (EV) subsidies, phasing out the sale of gas and coal boilers, changing product pricing to reflect how environmentally friendly products are, ensuring access to sustainable pension funds, increasing vegetarian/vegan options in public food provisioning, and higher taxes on red meat and dairy products. A description of the policies is provided in Table 2.

Two additional dependent variables were used to measure the level of conditional support for net zero policies if they would have potential lifestyle or potential financial implications, respectively. The questions were phrased as “If this policy meant that …, to what extent would you support or oppose it?.” The same response scale was used as for the main support for net zero policies variable. The phrasing of the questions for each of the eight net zero policies is shown in the Supplementary Information Table SI2.

##### Independent variables

The independent variables in this study included personal and policy-specific factors. The personal factors included *socio-demographics* (gender, age, ethnic background, country, type of area, deprivation), *climate change worry*, and *political values* (left vs. right wing orientation, liberal vs. authoritarian). The policy-specific variable included here was *perceived fairness* of the net zero policies (see below). A *framing* variable used dummies to indicate how net zero policies were presented to the respondents (i.e., the ‘neutral’ control text or a climate change, health or economic framing). This was done to control for any faming effects in the regression analyses.

*Gender* included the male and female categories; *age* was subdivided into three groups of 16–34 years, 35–54 years, and 55 years and older; *ethnic background* was subdivided into White (including white minority groups) and other ethnic backgrounds (which included Asian/Asian British. Black/Black British, and mixed, multiple, and other ethnic groups); *country* covered the four home nations (England, Scotland, Wales and Northern Ireland), with a separate English sub-category for London; and *area type* indicated whether respondents lived in a rural or urban area. All these socio-demographic groups were included as dummy variables. *Deprivation* reflected the Index of Multiple Deprivation (IMD) quintile. The latter variable was included as a continuous variable.

*Climate change worry* was measured by asking respondents “How worried are you about climate change?.” The response scale ranged from 1 (not at all worried) to 5 (extremely worried). The worry scale was adapted from (Poortinga et al., 2019). To explore the role of *political values* we adopted and measured two political values dimensions, i.e., a *‘left* versus *right’* and a *‘liberal* versus *authoritarian’* dimension. These constructs were measured using twelve attitude-style statements developed by Evans and colleagues (Evans et al., 1996), with six statements measuring the *‘left* versus *right’* dimension (“Ordinary working people get their fair share of the nation’s wealth,” “There is one law for the rich and one for the poor,” “There is no need for strong trade unions to protect employees’ working conditions and wages,” “Private enterprise is the best way to solve Britain’s economic problems,” “Major public services and industries ought to be in state ownership,” “It is the government’s responsibility to provide a job for everyone who wants one”) and six statements measuring the *‘liberal* versus *authoritarian’* dimension (“Young people today do not have enough respect for traditional British values” “Censorship of films and magazines is necessary to uphold moral standards,” “People should be allowed to organise public meetings to protest against the government,” “People in Britain should be more tolerant of those who lead unconventional lives,” “For some crimes, the death penalty is the most appropriate sentence, “People who break the law should be given stiffer sentences”). Respondents could use a 5-point scale ranging from 1 “strongly disagree” to 5 “strongly agree.” The two scales showed good internal consistency (Cronbach’s α of 0.73 and 0.74, respectively) and were only moderately inter-correlated (*r* = 0.36). The ‘left–right’ and ‘libertarian-authoritarian’ dimensions were standardised by transforming them into Z-scores.

*Perceived fairness* of net zero policies was measured with three items. Respondents were asked “How confident, if at all, are you that this policy would…” (a) give a fair outcome to everyone affected, (b) take into account the views of everyone affected, and (c) not be biased towards any one particular group. Respondents could respond using a 4-point scale ranging from 1 “Not at all confident” to 4 “Very confident.” The items were averaged and formed coherent scales for all eight policies (Cronbach’s α’s ranged between 0.84 and 0.88).

### Statistical analyses

First, descriptive analyses were used to show the extent to which the eight net zero policies were supported or opposed. Second, univariate (one-way) Analyses of Variance (ANOVAs) were conducted to examine whether the different framings have an impact on public support for the eight net zero policies. Where an overall ANOVA was significant, a Tukey’s HSD Test was conducted to see which framings were significantly different from the control group. Eta squared (η^2^) are reported for the effect sizes. Third, linear regression and multilevel modelling were used to examine factors in public support for the different net zero policies. Separate linear regressions were conducted for each policy, with policy support as the dependent variable. Multilevel modelling was used to examine factors in support across the eight net zero policies. In the multilevel analysis, support for the eight policies were seen as repeated measures (Level 1) within individuals (Level 2). This multilevel approach allows consistent effects of the factors to be estimated across the eight net zero policies. Independent variables included socio-demographics, climate change worry and political values variables, and perceived fairness of the policies. Finally, T-tests were conducted to test whether mentioning potential lifestyle and financial implications changes support for the net zero policies. Cohen’s d are reported for the effect sizes. All statistical analyses were conducted in RStudio v2021.09.0, with a number of R packages, including *tidyverse* (Wickham et al., 2019), *psych* (Revelle, 2022), and *lme4* (Bates et al., 2015).

## Results

### Support for net zero policies

**Figure 1:**
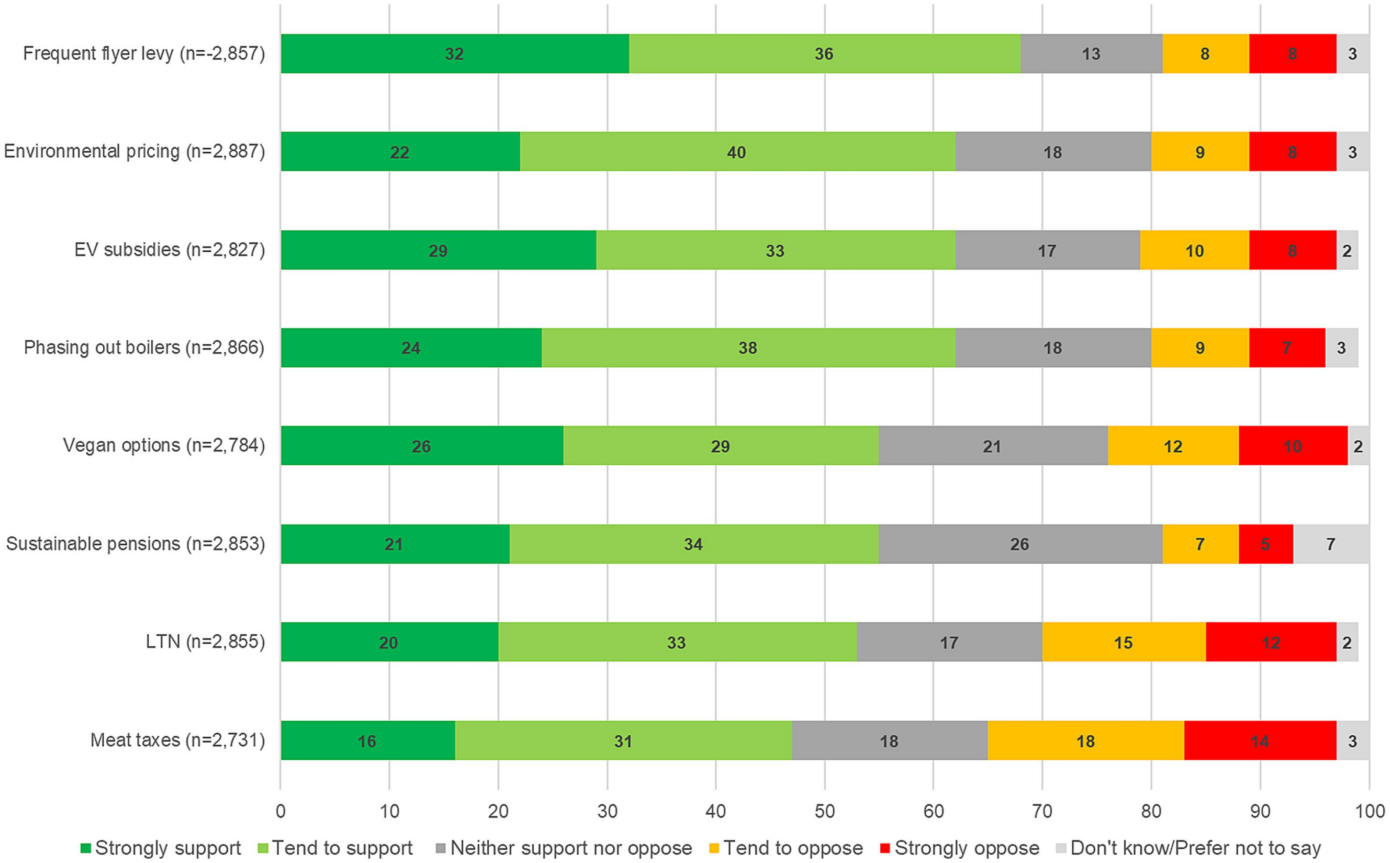
Support for eight net zero policies in the United Kingdom.

Figure 1 shows that there is widespread public support for the net zero policies that were included in the study, with low levels of opposition (also see Ipsos & CAST, 2022). Comfortable majorities support seven of eight policies presented. The highest levels of support were found for frequent flyer levies (68%), followed by changing product pricing to reflect how environmentally friendly products are, phasing out the sale of gas and coal boilers and electric vehicle subsidies (each 62%). Ensuring access to sustainable pension funds were supported by 55%. The results suggest that there is higher support for transport, energy and consumption-related policies than for food and diet-related policies, but further research with a wider range of polices is needed to solidify such a claim. The lowest levels of support in this study were for higher taxes on red meat and dairy products. Less than half of the population supported this policy. It is relevant to note that support (47%) was still higher than opposition (32%).

### Framing effects in support for net zero policies

**Figure 2:**
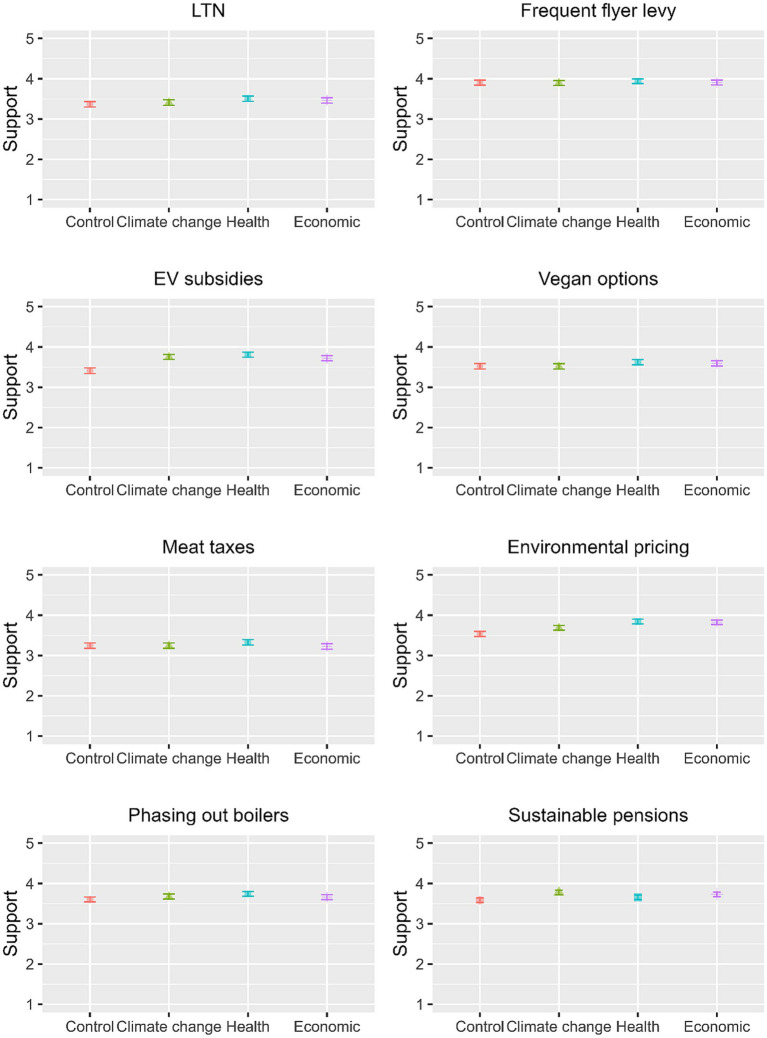
Mean support ratings for different framings of net zero policies.

Table 3 shows the mean support ratings and standard deviations for the different benefit framing of the eight net zero policies (also see Figure 2). The framing effects for EV subsidies [*F*(3, 278) = 14.500, *p* = 0.000, η^2^ = 0.015], environmental pricing [*F*(3, 2,767) = 11.040, *p* = 0.000, η^2^ = 0.012], and sustainable pensions [*F*(3, 2,673) = 3.928, *p* = 0.008, η^2^ = 0.004] were significant. Inspection of mean support ratings and the results of Tukey’s HSD post-hoc tests shows that all framings increased support for EV subsidies; that the health and economic (but not climate change) framings increased support for environmental pricing; and that only the health framing increased support for sustainable pensions. The framing effects for the other policies were non-significant [LTN: *F*(3, 2,784) = 1.528, *p* = 0.205, η^2^ = 0.002; Frequent flyer levy: *F*(3, 2,782) = 0.194, *p* = 0.901, η^2^ = 0.000; Vegan options: *F*(3, 2,792) = 1.163, *p* = 0.322, η^2^ = 0.001; Meat taxes: *F*(3, 2,773) = 0.850, *p* = 0.466, η^2^ = 0.001; Phasing out boilers: *F*(3, 2,775) = 1.597, *p* = 0.188, η^2^ = 0.002].

**Table 3:** Mean support (M) and standard deviations (SD) for different benefit framings of net zero policies.

	Framing condition				
Net zero policy	Control M (SD)	Climate change M (SD)	Health M (SD)	Economic M (SD)	Overall support M (SD)
Frequent flyer levy	3.90 (1.18)	3.89 (1.18)	3.94 (1.12)	3.9 (1.16)	3.91 (1.16)
Environmental pricing	3.53 (1.23)	3.69 (1.14)	3.84 (1.11)^Δ^	3.82 (1.07)^Δ^	3.72 (1.14)
Sustainable pensions	3.58 (1.07)	3.78 (1.11)^Δ^	3.66 (1.10)	3.73 (1.09)	3.69 (1.10)
EV subsidies	3.41 (1.35)	3.75 (1.19)^Δ^	3.81 (1.19)^Δ^	3.72 (1.22)^Δ^	3.67 (1.25)
Phasing out boilers	3.60 (1.17)	3.67 (1.22)	3.74 (1.14)	3.66 (1.19)	3.67 (1.18)
Vegan options	3.52 (1.30)	3.52 (1.29)	3.62 (1.28)	3.59 (1.26)	3.56 (1.28)
LTN	3.36 (1.30)	3.41 (1.32)	3.50 (1.27)	3.46 (1.26)	3.43 (1.29)
Meat taxes	3.24 (1.31)	3.24 (1.30)	3.33 (1.32) ^Δ^	3.22 (1.33)	3.26 (1.32)

^Δ^Mean differs significantly from the control description.

### Factors in support for net zero policies

Table 4 presents the results of the linear regression analyses for policy support. It shows that women express higher levels of support for increasing vegan options in public food provisioning and environmental pricing. It further shows that older age groups (and in particular those aged 55 and over) express higher levels of support for LTNs, frequent flyer levies, meat taxes, environmental pricing and phasing out boilers. Respondents with a non-white ethnic background express lower levels of support for LTNs, frequent flyer levies and environmental pricing.

**Table 4:** Factors in support for net zero policies.

	LTN	Frequent flyer levy	EV subsidies	Vegan options	Meat taxes	Environmental pricing	Phasing out boilers	Sustainable pensions	Overall^1^
	b (95% CI)	b (95% CI)	b (95% CI)	b (95% CI)	b (95% CI)	b (95% CI)	b (95% CI)	b (95% CI)	b (95% CI)
Constant	0.391^***^ (0.120, 0.662)	1.398^***^ (1.141, 1.656)	0.556^***^ (0.307, 0.805)	0.532^***^ (0.285, 0.779)	−0.095 (−0.341, 0.151)	0.581^***^ (0.348, 0.814)	0.700^***^ (0.463, 0.937)	1.191^***^ (0.956, 1.427)	0.627^***^ (0.516, 0.738)
Female	0.013 (−0.078, 0.104)	0.031 (−0.054, 0.116)	−0.042 (−0.128, 0.044)	0.166^***^ (0.084, 0.248)	0.081 (−0.002, 0.164)	0.181^***^ (0.104, 0.257)	−0.001 (−0.082, 0.080)	0.035 (−0.043, 0.114)	0.062^***^ (0.024, 0.100)
35–54	0.229^***^ (0.077, 0.381)	0.340^***^ (0.199, 0.481)	0.017 (−0.123, 0.158)	0.076 (−0.062, 0.213)	0.129 (−0.009, 0.266)	0.213^***^ (0.084, 0.341)	0.159^**^ (0.028, 0.290)	0.038 (−0.092, 0.167)	0.150^***^ (0.087, 0.213)
55 and over	0.264^***^ (0.117, 0.411)	0.452^***^ (0.313, 0.590)	0.024 (−0.113, 0.160)	0.119 (−0.016, 0.254)	0.147^**^ (0.013, 0.280)	0.314^***^ (0.189, 0.439)	0.165^**^ (0.037, 0.293)	0.046 (−0.081, 0.173)	0.194^***^ (0.133, 0.255)
Other ethnic background	−0.312^***^ (−0.522, −0.101)	−0.245^***^ (−0.427, −0.063)	0.034 (−0.149, 0.216)	0.125 (−0.049, 0.298)	−0.074 (−0.271, 0.123)	−0.205^**^ (−0.379, −0.030)	−0.083 (−0.257, 0.092)	−0.047 (−0.219, 0.126)	−0.082 (−0.166, 0.002)
Scotland	0.034 (−0.086, 0.153)	−0.015 (−0.123, 0.094)	−0.028 (−0.139, 0.084)	−0.113^**^ (−0.221, −0.006)	−0.130^**^ (−0.236, −0.024)	−0.120^**^ (−0.222, −0.017)	−0.091 (−0.195, 0.012)	−0.012 (−0.115, 0.091)	−0.056^**^ (−0.105, −0.007)
Wales	−0.159 (−0.386, 0.069)	−0.185 (−0.416, 0.046)	−0.042 (−0.253, 0.170)	−0.235^**^ (−0.449, −0.022)	0.014 (−0.197, 0.225)	−0.165 (−0.363, 0.033)	0.010 (−0.202, 0.222)	0.009 (−0.188, 0.207)	−0.102^**^ (−0.199, −0.005)
Northern Ireland	0.162 (−0.133, 0.456)	−0.281 (−0.567, 0.005)	0.199 (−0.067, 0.466)	−0.096 (−0.344, 0.151)	−0.144 (−0.387, 0.099)	0.026 (−0.200, 0.252)	0.031 (−0.240, 0.302)	−0.093 (−0.339, 0.152)	−0.039 (−0.158, 0.080)
England (London)	−0.249^***^ (−0.417, −0.080)	−0.149 (−0.310, 0.011)	0.094 (−0.061, 0.249)	0.035 (−0.122, 0.191)	0.119 (−0.041, 0.278)	−0.015 (−0.157, 0.128)	−0.050 (−0.200, 0.099)	0.001 (−0.143, 0.144)	−0.028 (−0.099, 0.042)
Area type	−0.025 (−0.130, 0.080)	−0.122^**^ (−0.220, −0.024)	0.100 (−0.001, 0.200)	0.025 (−0.070, 0.120)	0.016 (−0.080, 0.111)	−0.086 (−0.175, 0.003)	0.002 (−0.091, 0.096)	−0.036 (−0.127, 0.055)	−0.014 (−0.058, 0.030)
Neighbourhood deprivation	0.014 (−0.019, 0.048)	0.018 (−0.014, 0.049)	−0.042^**^ (−0.074, −0.010)	−0.045^***^ (−0.075, −0.014)	−0.021 (−0.053, 0.010)	−0.030^**^ (−0.058, −0.001)	0.011 (−0.019, 0.041)	0.010 (−0.019, 0.040)	−0.012 (−0.026, 0.002)
Climate change worry	0.306^***^ (0.256, 0.356)	0.280^***^ (0.234, 0.326)	0.381^***^ (0.335, 0.427)	0.336^***^ (0.291, 0.381)	0.394^***^ (0.347, 0.441)	0.412^***^ (0.369, 0.455)	0.376^***^ (0.332, 0.421)	0.335^***^ (0.291, 0.379)	0.351^***^ (0.330, 0.371)
Left–right scale	−0.089^***^ (−0.140, −0.039)	−0.125^***^ (−0.172, −0.078)	−0.085^***^ (−0.133, −0.036)	−0.075^***^ (−0.122, −0.029)	−0.080^***^ (−0.127, −0.034)	−0.031 (−0.073, 0.011)	−0.063^***^ (−0.107, −0.019)	−0.121^***^ (−0.166, −0.077)	−0.085^***^ (−0.106, −0.064)
Libertarian-authoritarian scale	−0.113^***^ (−0.164, −0.062)	−0.079^***^ (−0.126, −0.032)	−0.126^***^ (−0.175, −0.077)	−0.219^***^ (−0.266, −0.173)	−0.182^***^ (−0.229, −0.135)	−0.188^***^ (−0.231, −0.145)	−0.161^***^ (−0.205, −0.116)	−0.147^***^ (−0.192, −0.103)	−0.153^***^ (−0.175, −0.132)
Perceived fairness	0.836^***^ (0.767, 0.905)	0.631^***^ (0.566, 0.695)	0.699^***^ (0.637, 0.761)	0.762^***^ (0.702, 0.822)	0.876^***^ (0.813, 0.938)	0.598^***^ (0.538, 0.657)	0.696^***^ (0.635, 0.757)	0.571^***^ (0.511, 0.632)	0.721^***^ (0.696, 0.746)
Climate change framing	−0.050 (−0.176, 0.076)	−0.062 (−0.180, 0.056)	0.193^***^ (0.073, 0.312)	−0.084 (−0.198, 0.031)	−0.040 (−0.156, 0.077)	0.130^**^ (0.023, 0.236)	0.063 (−0.048, 0.175)	0.106 (−0.003, 0.215)	0.033 (−0.019, 0.086)
Health framing	0.099 (−0.027, 0.225)	−0.008 (−0.127, 0.112)	0.353^***^ (0.233, 0.473)	0.073 (−0.041, 0.186)	0.056 (−0.060, 0.172)	0.231^***^ (0.125, 0.337)	0.136^**^ (0.023, 0.249)	−0.009 (−0.119, 0.101)	0.111^***^ (0.058, 0.164)
Economic framing	0.075 (−0.051, 0.202)	−0.099 (−0.217, 0.020)	0.182^***^ (0.062, 0.302)	−0.007 (−0.122, 0.108)	−0.083 (−0.200, 0.034)	0.208^***^ (0.101, 0.315)	0.031 (−0.081, 0.144)	0.066 (−0.045, 0.177)	0.043 (−0.011, 0.096)
Observations	2,141	2,093	2,128	2,136	2,159	2,129	2,099	1,962	16,847

^**^p < 0.05; ^***^p < 0.01; ^1^the overall effects were estimated using multilevel modelling; *b* = unstandardised regression coefficients; 95% CI = 95% confidence interval.

Table 4 also shows that support for the net zero polices vary across different geographical areas. Respondents living in Scotland express less support for increasing vegan options in public food provisioning, meat taxes and environmental pricing; respondents living in Wales express less support for increasing vegan options in public food provisioning; and respondents living in London shows less support for LTNs as compared to those living in England (outside of London). Respondents from urban areas express lower levels of support for frequent flyer levies than those from rural areas, while respondents from more deprived neighbourhoods express lower levels of support for EV subsidies, increasing vegan options in public food provisioning and environmental pricing than those from less deprived areas.

In terms of the psychological variables, climate change worry is the most consistent personal factor in support for the net zero policies: those with higher levels of worry about climate change have higher levels of support for all net zero policies. In terms of political values, more right-wing individuals have lower levels of support than more left-wing individuals for all net zero policies except environmental pricing, while respondents with more authoritarian views express less support for net zero policies than those with more liberal views. The strongest effects were found for perceived fairness. That is, those who perceive a net zero policy to be fair express higher levels of support for them, as compared to those who do not perceive a policy to be fair.

The regression analyses also included the framing variable indicating how the net zero policies were presented to the respondents, showing whether there is a framing effect when all personal and policy-specific factors are controlled for. The results depart slightly from the univariate analysis of variance reported above, in that no significant effects were found for meat taxes and for sustainable pensions. Conversely, and also in contrast to the univariate ANOVA results, the health framing was found to increase support for phasing out gas/coal boilers. Largely in line with the univariate ANOVA results, all framing conditions (i.e., climate change, health, and economic) were found to increase support for EV subsidies and for environmental pricing.

The final column of Table 4 presents the results of the multilevel model with policy support ratings (Level 1) nested within individuals (Level 2). The results suggest that women and older age groups have higher levels of support for net zero policies overall. Respondents from Scotland and Wales express slightly lower level of support for net zero policies than those from England. In line with the individual regression analyses, respondents with higher levels of worry about climate change express more support across the eight net zero policies; respondents with more right-wing and authoritarian views express less support for net zero policies; and perceived fairness was the strongest predictor of support across the eight net zero policies. The results further suggest that a health framing may increase support across the eight net zero policies.

### Support for net zero policies with potential lifestyle and financial implications

Table 5 shows mean support for the eight net zero policies with and without mentioning potential lifestyle and financial implications. It is clear that mean support for the net zero policies is lower when potential personal implications of these policies are mentioned. The only exception is Environmental pricing, for which support is higher when potential lifestyle implications are mentioned, t(2718) = −15.87, *p* < 0.001 (Cohen’s *d* = 0.28). For all other cases support decreased when mentioning potential lifestyle implications (Frequent flyer levy, t(2394) = 32.239, *p* < 0.001, Cohen’s d = 0.56; Sustainable pensions, t(2308) = 22.289, *p* < 0.001, Cohen’s *d* = 0.50; EV subsidies, t(2599) = 18.769, *p* < 0.001, Cohen’s *d* = 0.30; Phasing out boilers, t(2573) = 24.434, *p* < 0.001, Cohen’s *d* = 0.35; Vegan options, t(2690) = 23.553, *p* < 0.001, Cohen’s *d* = 0.30; LTN, t(2612) = 21.106, *p* < 0.001, Cohen’s *d* = 0.32; and Meat taxes, t(2674) = 13.481, *p* < 0.001, Cohen’s *d* = 0.20). Mentioning potential financial implications decreased support for all eight net zero policies [Frequent flyer levy, t(2433) = 36.942, *p* < 0.001, Cohen’s *d* = 0.68; Environmental pricing, t(2738) = 12.450, *p* < 0.001, Cohen’s *d* = 0.20; Sustainable pensions, t(2450) = 55.437, *p* < 0.001, Cohen’s *d* = 1.21; EV subsidies, t(2591) = 33.450, *p* < 0.001, Cohen’s *d* = 0.56; Phasing out boilers, t(2573) = 39.257, *p* < 0.001, Cohen’s *d* = 0.64; Vegan options, t(2715) = 42.943, *p* < 0.001, Cohen’s *d* = 0.70; LTN, t(2710) = 47.340, *p* < 0.001, Cohen’s *d* = 0.86; and Meat taxes, t(2675) = 17.860, *p* < 0.001, Cohen’s *d* = 0.27].

**Table 5:** Mean support (M) and standard deviations (SD) for net zero policies with and without potential lifestyle and financial implications.

Net zero policies	Mean support without lifestyle/financial implications M (SD)	Mean support with lifestyle implications M (SD)	Mean support with financial implications M (SD)
Frequent flyer levy	3.91 (1.16)	3.21 (1.32)	3.07 (1.31)
Environmental pricing	3.72 (1.14)	4.02 (1.00)	3.49 (1.17)
Sustainable pensions	3.69 (1.10)	3.12 (1.16)	2.31 (1.18)
EV subsidies	3.67 (1.25)	3.29 (1.27)	2.94 (1.34)
Phasing out boilers	3.67 (1.18)	3.24 (1.30)	2.88 (1.30)
Vegan options	3.56 (1.28)	3.16 (1.42)	2.64 (1.34)
LTN	3.43 (1.29)	3.01 (1.31)	2.34 (1.24)
Meat taxes	3.26 (1.32)	2.99 (1.41)	2.90 (1.38)

## Discussion

### Summary of results

Meeting ambitious carbon reduction targets to keep climate change within safe limits requires transformative change to society. The transformation to net zero needs to be accelerated by public policy to ensure the reductions are made in time (Roberts et al., 2018). This needs to involve policies that support behaviour change across a range of activities and domains to reduce carbon emissions (Nielsen et al., 2020), as technological change alone is not sufficient (Akenji et al., 2021).

This study provides new insights into public support for net zero policies in the UK, by exploring factors and framing effects across a range of net zero policies. In particular, it considered personal and policy-specific factors in support for eight policies that are already enacted or considered in the UK in a number of behavioural domains, and examined whether framing net zero policies in terms of their benefits can help to increase public support for them. Furthermore, the study explored whether mentioning potential cost and lifestyle implications of net zero policies can affect support for them.

The study found more support than opposition for all eight net zero policies. The highest levels of support were found for frequent flyer levies, environmental product pricing, phasing out the sale of gas and coal boilers, and electric vehicle subsidies. The lowest levels of support were found for taxes on meat and dairy products. This was the only policy with less than 50% support among the UK public. Relatively low levels of support were also found for LTNs and increasing vegetarian and vegan options in public food provisioning.

These results are in line with other research in the UK and elsewhere showing that the public understand the importance of policies targeting transport, energy and material consumption behaviours (Steentjes et al., 2021). However, they tend to underestimate carbon emissions from food (Wynes et al., 2020) and often do not see the necessity for change in this area (Steentjes et al., 2021). This may at least partly explain why there is lower support for higher taxes on red meat and dairy products, although the label ‘tax’ is also known to decrease support compared to equivalent concepts, such as ‘levy’ (Baranzini and Carattini, 2017). A repeat of the survey conducted in Autumn 2022 shows that support for the net zero policies has remained high despite the cost-of living-crisis. Some policies even enjoyed higher support in 2022 than in 2021 (see Supplementary Information Table SI3).

The study further demonstrated that framing net zero policies in terms of their co-benefits can improve public support, but the effects are quite small. The health framing was particularly effective in increasing public support for net zero policies, with some effects found for the economic and climate change framings as well. It should be noted that the co-benefit frames did not increase support for four out of eight policies (i.e., frequent flyer levies, phasing out boilers, increasing vegetarian/vegan options, and LTNs). It may be that some of these policies are already associated with specific co-benefits, which might explain why the different framings were not very successful in increasing support. The current evidence on the effects of co-benefits framing is mixed. While some research has found consistent effects across a large number of countries (Bain et al., 2016), others conclude that an alternative framing or justification for climate policy is unable to increase public support for climate mitigation (Bernauer and McGrath, 2016; Fesenfeld and Rinscheid, 2021). If there are any effects, they are likely to be small and have to compete with counter frames that are present in public and media discourses (Boykoff, 2014; O’Neill et al., 2015; Stoddart et al., 2023).

The results suggest that spelling out policy costs may have a greater effect than mentioning the co-benefits. This may be because of the direct cost implications (making the policies less attractive) or because mentioning the implications may make the policy more personally relevant. Previous research shows that even mentioning a very modest cost can reduce climate policy support (Whitmarsh et al., 2019). This asymmetry in cost–benefit framing is in line with the well-established prospect theory (Kahneman and Tversky, 1979) and has important implications for communication (see below). The one exception is environmental pricing, for which support is higher when potential lifestyle implications are mentioned. A possible explanation for this surprising effect is that people had already taken into account those potential implications of the policy.

A series of regression analyses showed that there were some differences in support for the eight net zero policies across different socio-demographic groups, but the effects were not consistent. Overall, net zero policies were found to be more supported by women and older age groups. Other notable results were that that LTNs are supported less in London, and that certain net zero policies are supported less by minority ethnic backgrounds living in more deprived neighbourhoods. These inconsistent findings may be because demographic factors only partially map on to people’s needs and abilities, with more direct effects on policy support from perceived policy fairness (*cf.* Player et al., 2023).

Stronger effects were found for individual values and beliefs than for socio-demographic factors, with effects also more consistent across the eight net zero policies. That is, people who are worried about climate change – perhaps unsurprisingly – express higher levels of support for all eight policies, as do those with more left-wing (versus right wing) and libertarian (versus authoritarian) political values. These results are in line with a substantial body of empirical and theoretical research showing that problem perception (climate concern) is one of the most important determinants of (climate) policy support (Poortinga et al., 2012; Drews and van den Bergh, 2016; Bouman et al., 2020; Bergquist et al., 2022); and that political orientation (e.g., left versus right, liberal versus conservative) shapes beliefs about climate in particular in anglophone countries (Hornsey et al., 2018; Poortinga et al., 2019), including for climate policies (Poortinga et al., 2012, 2022; Ejelöv and Nilsson, 2020; Bergquist et al., 2022).

The strongest and most consistent predictor of net zero support was, however, perceived fairness. The results fit well with the literature showing perceived fairness is a key driver of policy support (Maestre-Andrés et al., 2019; Climate Assembly UK, 2020; Ejelöv and Nilsson, 2020; Liu et al., 2020; Bergquist et al., 2022). Our measure of perceived fairness encompasses distributional and procedural elements, both of which are known to be important for policy support; but our other findings suggest wider interpretations of fairness may be at play. The relative popularity of frequent flyer levies and environmental fee-dividend pricing may be due to these policies reflecting the ‘polluter pays’ principle (i.e., fair distribution of costs) but also conserving freedom of choice; while our findings that personal cost reduces support may also be taken as form of perceived unfairness for the individual (Webster et al., 2022). Notably, perceptions of fairness may be constructed post-hoc to justify or reinforce intuitive policy preferences; that is, people inclined to support net zero policies (e.g., due to climate concern) may align their policy evaluations (e.g., of fairness) with this support (Zajonc, 1980).

### Strengths and limitations

The main strength of the current study is that it made use of a high-quality probability sample collected by a professional social survey company (Ipsos, 2022). The sample is large enough to be able to partition the survey to help reduce the burden on the respondents and thus to maintain response quality. Random probability sampling is the gold standard of survey research and can be used to make robust population and regression estimates (Thompson, 2012). The wide range of policies examined was also a strength and point of novelty. A further strength of the study is that it used policies that are already enacted or proposed to be used in the UK. This was done so that participants could easily envisage them and thus have or form an opinion about them, but also to ensure that the results of the study can be used by policymakers who are responsible for designing and implementing net zero.

As any study, it also has a number of limitations. It was for example not possible to include a larger number of policies. Ideally, follow-up research would include different types of policies (e.g., subsidies, taxes, regulation, information) systematically across the different behavioural domain to be able to reliably apportion variance in support to those different policy characteristics (*cf.*, Poortinga et al., 2022). This would be able to show for example whether ‘pull’ measures are always preferred over ‘push’ measures (Steg et al., 2006), or whether taxes are more acceptable in certain behavioural domains but not in others. Carbon taxes are generally disliked (Maestre-Andrés et al., 2019; Umit and Schaffer, 2020), but frequent flyer levies, also a tax, was the most supported policy in this study. Other limitations relate to the framing and the questions to determine the impacts of potential cost and lifestyle implications of net zero policies. The specific effects depend on the way information is presented, and it is therefore possible that the relatively small framing effects are because the frames are not powerful enough (*cf.*, Bernauer and McGrath, 2016). In contrast, the potential implications used to determine the conditional support for the different net zero policies (see Supplementary Information Table SI2) may never materialise. Nevertheless, results showing high levels of support based on generic policy descriptions may miss the nuances and conditionality of such support (Demski, 2011). A related limitation was that the study only allowed the inclusion of a limited number of co-benefit frames. A main finding of Bain et al. (2016) was that the social benefits (‘benevolence’) can motivate action on climate change. It may therefore be interesting to include such social benefits of climate policy to cover all pillars of sustainable development. The inclusion of social co-benefits of action may help strengthen the results showing that perceived fairness as the most important predictor of net zero policy support. It seems to us that this is a limitation of the study that should be discusses.

## Conclusion and policy implications

The main finding of this study is that the UK public are generally supportive of net zero, with the results showing that there is a high level of support for a variety of policies that can be used to establish change in the behavioural domains of travel, diet, material and energy consumption, and financial investment. The public appear to support net zero policies slightly more if they are framed in terms of their health, economic and climate change benefits, but these effects are small. This suggests that, in line with other research, a simple reframing is unlikely to lead to massive changes in support, although it may still be used to create awareness of the wider benefits of net zero policies among less committed publics (Bain et al., 2016).

While support for net zero appears robust, results from this study suggest that this may drop sharply when potential cost implications are presented. This may mean that public support for net zero policies is potentially fragile. Policymakers need to understand the that where the general public have concerns about the cost and lifestyle implications of net zero policies, support for these policies may change. Simply ignoring potential personal costs when introducing policies is likely to create problems when these come to light.

Perceived fairness has been found to be the strongest predictor of policy support, and the way costs and lifestyle implications (are perceived to) play out across different groups is likely to be a core factor in net zero policy support. Indeed, arguments against – for example – ultra-low emission zones (ULEZs) and low-traff eighbourhoods (LTNs) are often phrased in terms of how unfair they are for specific groups (BBC, 2023a,b). Even if distributional effects may be counter to the claims made by those opposing the policy (Aldred et al., 2021; Yang et al., 2022), such arguments can be persuasive and thus lead to lower support (see, e.g., Ipsos & CAST, 2022).

The overall conclusion is that the public are supportive of net zero polies in the UK, but that this support cannot be taken for granted. The public are more likely to be supportive of net zero if and when they feel they are fair in terms of the decision-making process and their outcomes. A comprehensive and inclusive public engagement strategy may be needed that takes into account citizens’ needs and concerns to build a mandate for action (Verfuerth et al., 2023). This may help to avoid public backlash when policies are implemented.

## Data availability statement

The raw data supporting the conclusions of this article will be made available by the authors, without undue reservation.

## Ethics statement

The studies involving humans were approved by School of Psychology Research Ethics Committee. The studies were conducted in accordance with the local legislation and institutional requirements. The participants provided their written informed consent to participate in this study.

## Author contributions

WP: Conceptualization, Writing – original draft, Writing – review & editing, Formal analysis. LW: Conceptualization, Writing – original draft, Writing – review & editing. KS: Writing – review & editing, Conceptualization. EG: Conceptualization, Data curation, Project administration, Writing – review & editing. ST: Conceptualization, Data curation, Project administration, Writing – review & editing. RB: Conceptualization, Data curation, Project administration, Writing – review & editing.
