# Supplementary material for: Factors and framing effects in support for net zero policies in the United Kingdom
Source: Front Psychol. 2023 Dec 15;14:1287188. doi: 10.3389/fpsyg.2023.1287188 (PMC10758422; doi:10.3389/fpsyg.2023.1287188)
Supplement: Supplementary file 1 [file Data_Sheet_1.pdf]

Supplementary Information

Table SI1: Descriptions of the climate change, health and economic framing conditions for the net zero policies.

	Framing condition		
Net zero policies	Climate Change	Health	Economic
LTN	This would help to tackle climate change, as petrol and diesel vehicles are a major contributor to carbon emissions. Reducing the number of vehicles on our roads will reduce the level of carbon emissions in our atmosphere.	This would help to improve the health of people living and working in the area, as fewer vehicles on our roads will reduce the level of air pollution and could encourage people to take exercise by walking or cycling rather than travelling by car. It could also reduce the amount of traffic – and accidents – on our roads.	This would benefit local businesses, as customers are able to access shops more easily on foot, and local cafes may be able to use street space for outside tables.
Frequent flyer levy	This would help to tackle climate change, as flying is a very polluting form of transport and fewer planes in the air will significantly reduce the level of carbon emissions in our atmosphere.	This would help to cut noise and air pollution for people living near airports, by reducing the number of flights taken. This would mean health improvements for these local communities.	This would help to make other forms of long-distance transport such as trains or ferries more competitive and affordable. This would also create jobs in the local UK tourist sector if more people choose to holiday locally instead of traveling abroad.
EV subsidy	This would help to tackle climate change, as driving a car powered by fossil fuels, like petrol or diesel, causes a large amount of carbon emissions. Electric vehicles can reduce these emissions and help significantly cut drivers' carbon footprint.	This would help to improve air quality in towns and cities because petrol and diesel vehicles cause air pollution that affect adults' and children's health. Electric vehicles cause less air pollution, so reduce the risks of asthma, heart disease and lung cancer for people living and working in towns and cities.	This would help to cut motorists' bills, as electric vehicles are much cheaper to run than petrol or diesel cars. By increasing the sales of these vehicles, this policy would also help reduce how much it costs to make electric vehicles so prices go down further, and others can afford them.
Vegan options	This would help to tackle climate change, as red meat and dairy are the most polluting types of food to produce, while the production of vegetarian and vegan foods	This would help to improve the health of hospital patients, school children, and public sector workers, as cutting down on red meat and eating more vegetarian and vegan	This would help to make vegetarian and vegan food producers more competitive and create jobs in this sector. By providing an initial customer base for these

	tends to emit much lower levels of greenhouse gases.	foods can cut the risk of heart disease and certain types of cancer.	products, it could also help reduce their price so others can afford them.
Meat taxes	This would help to tackle climate change, as red meat and dairy are the most polluting types of food to produce, while the production of vegetarian and vegan foods tends to emit much lower levels of greenhouse gases.	This would help to improve the health of people who change their eating habits as a result, as cutting down on red meat and increasing vegetarian and vegan alternatives in people's diets can cut their risk of heart disease and cancer.	This would help to make vegetarian and vegan food producers more competitive and create jobs in this sector. By providing an initial customer base for these products, it could also help reduce their price so others can afford them.
Environmental pricing	This would help to tackle climate change, as reducing the sale of products that use a lot of energy or materials will reduce the level of carbon emissions in our atmosphere.	This would help to preserve resources that are becoming rarer. It would also help reduce the amount of rubbish going to landfill by encouraging more recycling of materials by businesses and people.	This would help to make locally manufactured and recyclable products more competitive and create jobs in this sector. By providing an initial customer base for these products, it could also help reduce their price so others can afford them.
Phasing out boilers	This would help to tackle climate change, as heating systems powered by fossil fuels are one of the most significant sources of carbon emissions. Renewable energy alternatives, like electric heat pumps, are very low carbon and more.	Technologies like electric heat pumps require very little maintenance and tend to be more reliable and safer than gas boilers. In addition, heat pumps are versatile since they can be used to provide cooling in warmer weather, as well as heating in the winter.	Technologies like electric heat pumps are much cheaper to run than gas boilers, so can significantly cut householders' energy bills. This can particularly help poorer households who spend a larger proportion of their income on energy bills.
Sustainable pensions	This would help to tackle climate change as many pensions are invested in companies that have a negative effect on the environment, such as fossil fuels and deforestation. By making sustainable pensions a default option for the public, investments in initiatives that harm the planet would decline, and investments that	Most people are not aware of the impact their pensions have on people or the planet. Even when sustainable pensions are provided as an option, people may not be aware enough to opt in. By making sustainable pensions a default option, it would ensure more people would sign up without the hassle, energy, time and knowledge required to do this themselves.	This would help to make better returns on investments, as sustainable investments have recently been shown to outperform standard investment options. Sustainable investments are therefore the more financially sound choice as they show the greatest return.

were better for the planet
would increase.

Table SI2: Potential personal lifestyle and financial implications of the eight net zero policies ("If this policy meant that …, to what extent would you support or oppose it?").

	Potential implication	
net zero policies	Lifestyle	Financial
LTN	…you personally were not able to drive in certain areas – unless you lived or worked there.	…you personally had to pay more council tax.
Frequent flyer levy	…you personally were not able to take flights abroad very often.	…you personally had to pay more to take a flight.
EV subsidy	…you personally had a more limited range to choose from when buying a car.	…you personally had to pay more to drive your petrol/diesel car.
Vegan options	…you personally were not able to eat as many meat and dairy products in these settings.	…you personally had to pay higher taxes to fund this policy.
Meat taxes	…you personally were not able to eat as many meat and dairy products as you do now.	…you personally had to pay more for meat and dairy products.
Environmental pricing	…you personally were not able to buy as much of certain products e.g., single-use plastics as you do now.	…you personally had to pay more for some products.
Phasing out boilers	…you personally were not able to install a new gas or coal boiler in your home – and had to install an alternative heating system instead.	…you personally had to pay more to install an alternative heating system in your home.
Sustainable pensions	…you personally had to opt out of a sustainable pension fund if you wanted to save in a regular pension fund.	…you personally may get a smaller return from your pension savings.

Table SI3: Support for eight net zero policies in the UK in Autumn 2022.

	Strongly support	Tend to support	Neither support nor oppose	Tend to oppose	Strongly oppose	Don't know/Prefer not to say
Meat taxes	17%	30%	16%	20%	16%	1%
LTN	20%	30%	16%	19%	15%	1%
Sustainable pensions	22%	33%	27%	8%	6%	4%
Vegan options	26%	29%	18%	14%	13%	1%
Phasing out boilers	17%	32%	20%	16%	13%	2%
EV subsidies	18%	27%	16%	19%	18%	1%
Environmental pricing	20%	40%	17%	12%	10%	2%
Frequent flyer levy	31%	35%	14%	10%	8%	1%

Note: Sample size is n=16,160; data were collected from 27 October to 2 November 2022 using Ipsos' UK Knowledge Panel; the percentages do not always add up to 100% due to rounding.
